# Feasibility first: Expanding access before fixing learning^☆^

**DOI:** 10.1016/j.ijedudev.2023.102949

**Published:** 2024-01

**Authors:** Lee Crawfurd

**Affiliations:** Center for Global Development, UK

**Keywords:** Secondary education, Learning outcomes, School access, Education policy, Developing countries

## Abstract

The first SDG4 target is to both expand access to universal secondary school and to ensure that all children achieve minimum learning outcomes in primary school. To the extent that action must be prioritized, this article argues that achieving universal secondary education is both more feasible at scale and has clearer benefits than improving learning outcomes in primary schools in developing countries. Removing barriers to access such as fees and exam requirements has been demonstrated to have consistent impacts at scale, even in contexts of weak state capacity. By contrast, efforts to improve school quality have been much less consistent. Wage gains from secondary schooling have been demonstrated repeatedly, even in contexts of low quality education. Wage gains from improving primary school quality have not. Governments should thus focus on reforms that reliably expand access, rather than complex interventions to improve learning that often fail at scale.

## Highlights

• SDG4 targets expanded access to and quality of school.
• Universal secondary is more feasible than improving quality of primary.
• Removing barriers to access has been shown to work at scale.
• We should focus on reforms that work reliably not complex programs that don’t scale.

## 1 Introduction

The Sustainable Development Goals (SDGs) are expansive. The first of ten education goals is “By 2030, ensure that all girls and boys complete free, equitable and quality primary and secondary education leading to relevant and effective learning outcomes.” Despite this, the World Bank and other international actors have argued for a focus on prioritizing efforts to resolve the “global learning crisis” in literacy and numeracy (Pritchett, 2013, World Bank, 2018). That less than half of ten-year-olds in low-income countries can read and understand a simple text is a stunning fact that demands attention. Yet despite the depth of the learning crisis, the shift in policy focus to narrow measures of early grade test scores may not be the most effective way improving educational outcomes and making progress on the Sustainable Development Goals (SDGs). Instead, if we are to prioritise, it may be more effective to first finish the access agenda, at both primary and secondary level. This goal is feasible, politically attractive, and just needs investment.

Whilst there has been rapid progress since 2000, primary school completion rates in low and middle income countries have plateaued at around 90%, and lower secondary school completion around 75% (UNESCO Institute for Statistics, 2023). Significant barriers persist, especially at secondary level, where fewer than half of sub-Saharan African countries have tuition-free access (Gruijters et al., 2023). Evidence shows each extra year of schooling delivers substantial earnings gains, on average around 10% (Montenegro and Patrinos, 2021). By contrast, proof is lacking that efforts to improve learning outcomes reliably succeed at scale (Crawfurd et al., 2022b). Whilst there is evidence that some targeted interventions can improve learning (Angrist et al., 2023), this evidence comes from studies conducted at relatively small scale.

This piece thus argues governments should focus first on simple, scalable reforms to expand enrollment, chiefly abolishing school fees and exam barriers. More complex efforts to transform classroom teaching practices to improve learning, whilst important, often fail to work at national scale and exhibit uncertain returns. This access agenda - getting kids into school - is still far from complete.

In Section 2 I discuss in more detail the context of a new transatlantic consensus on foundational literacy and numeracy as priority goals. In Section 3 I discuss the evidence on the economic returns to investments in school access and quality. In Section 4 I elaborate on evidence around the feasibility of expanding access and improving quality at scale through government systems. In Section 5 I discuss the relative costs of alternative strategies, and in Section 6 I conclude.

## 2 The learning crisis and the new transatlantic consensus

The Millennium Development Goals (MDGs), established in 2000, prioritized universal primary enrollment as the education goal. However, with the adoption of the SDGs in 2015, priorities shifted based on growing evidence of a "learning crisis" - that many children in school were not acquiring foundational literacy and numeracy skills. This prompted a new focus on measuring and improving learning outcomes, not just access. The SDG agenda for education is also much broader though than the MDG agenda was. The SDG education goals include access to early childhood development, vocational education, eliminating gender disparities, and enhancing inclusion of vulnerable groups. The first target alone pulls in two slightly different directions - it calls for universal completion of secondary school, but also a focus on quality, or measured learning outcomes. International donors are increasingly focused on foundational literacy and numeracy. International aid for schools (or ‘basic education’) is dominated by the United States and United Kingdom (other large donors to education such as Germany and Japan provide more financing for universities and vocational training). The focus of the US and UK has narrowed in recent years to improving “foundational literacy and numeracy” - crystallised in the World Bank’s “learning poverty” indicator, and latterly included as a SDG indicator. Surely no one could be against such a goal. The challenge is in how to achieve it. Despite all this new attention on learning, there is little evidence of progress.

This new transatlantic consensus on foundational literacy and numeracy hasn’t yet caught on in developing countries. This matters given that most education spending is national and not aid-funded. Analysis of national statements of commitment from 100 + countries presented at the 2022 UN Transforming Education Summit, shows that whilst “teaching and learning” are high priorities, very few countries made specific reference to the “learning crisis” or “foundational literacy and numeracy” (Crawfurd et al., 2022a). Similarly, interviews with policymakers from 35 developing countries in 2021 found that they placed a higher priority on socialisation and school completion as objectives than achieving universal foundational skills (Crawfurd et al., 2021).

## 3 The economic benefits are clearer from access

The “Mincerian” returns to schooling are one of the most consistent and robust findings in social science. Across hundreds of datasets from hundreds of countries, the average wage gain from an additional year of school is around 10% (Montenegro and Patrinos, 2021). This is the case in both rich and poor countries alike (on average 9.8% in high-income and upper-middle income countries, and 9.9% in low-income and lower-middle income countries). These observational gains may of course be confounded by endogenous selection into schooling, but they are supported by quasi-experimental studies with a much stronger claim to causal inference, that show similar results (see for example Duflo, 2001 and Khanna, 2023a, though noting that the robustness of these results has been challenged by Roodman, 2023, Roodman, 2022, with response from Khanna, 2023b).

There are other important benefits from schooling alone (even poor quality schooling) - not least child mortality. Experimental and quasi-experimental evidence shows that each additional year of school a girl receives reduces their chance of seeing their child die by 1–2% (Andriano and Monden, 2019, Duflo et al., 2021, Grépin and Bharadwaj, 2015). This effect comes partly through learning (Kaffenberger and Pritchett, 2021, Kaﬀenberger et al., 2018) but partly also simply through slightly delaying childbirth until girls are older (Perez-Alvarez and Favara, 2023).

By contrast, there is a striking lack of comparable evidence for wage gains from achieving basic literacy and numeracy. Evidence we do have suggests that an improvement in skills by around 0.25 standard deviations (a typical effect size of an early grade reading program) is associated with around 4% higher earnings (Glewwe et al., 2022). Cross-country approaches also suggest that quality matters (Hanushek, 2017), however these observational macro approaches are less credible than the clearer micro evidence.

There are of course spillovers between the two approaches - increasing access to secondary incentives children do better on primary school tests (Sandholtz, 2022), and vice-versa improving learning in primary school may enable children to progress to secondary school (Evans and Hares, 2021, Zuilkowski et al., 2016). But marginal funding and policy attention needs to decide between the two.

One recent modelling approach directly considers the economic gains from increasing access to secondary school versus improving quality in primary school (Fujimoto et al., 2023). Whilst the authors favour investment in quality over access to secondary school, their approach actually suggests that there are similar welfare gains from both approaches. Crucially, they note that removing fees alone is insufficient to boost secondary enrolment, but that removing both fees and test barriers to secondary school performs similarly in welfare terms (of around 5%) to implementing policies that achieve a nationwide improvement in school quality raising test scores by 0.1 standard deviations.

## 4 The need for feasibility

Addressing access is expensive but easier to achieve. Whilst improving learning in primary or expanding access to secondary might have similar welfare benefits, removing fees and arbitrary test barriers is just very clearly much easier to achieve, and can be done almost at the stroke of a pen. For example, universal primary education policies in Ethiopia, Malawi, and Uganda, all increased schooling attainment, with follow-on reductions in teenage births and marriages (Moussa and Omoeva, 2020) . Fees remain an important barrier in many countries - free secondary education is available only half of African countries (Gruijters et al., 2023), and even where tuition fees are officially banned, many households continue to have to pay informal fees for tuition. High-stakes exams at the end of primary school may account for a quarter of drop-out at the end of primary school (Rossiter and Konate, 2023). By contrast improving quality is less about spending more money than about changing the behaviour of thousands of teachers across a country, to improve their teaching ability. This characterisation of the differences between learning and access as challenges is supported by policymakers. In another CGD survey, policymakers said that the main barrier to improving learning was implementation capacity rather than financing, whereas the main barrier to expanding access to secondary school was financing and not implementation capacity.

An alternative to comparative cost-effectiveness analysis is identifying case studies of success. Examples include the Bill and Melinda Gates Foundation “Exemplars in Global Health”, in which positive outliers are scrutinised for lessons (Exemplars, 2023). The Millions Saved project from the Center for Global Development is similar (Glassman and Temin, 2016). There are few case studies of success in improving learning at scale. The World Bank has compiled data from a range of different international assessments, finding that in contrast to the rapid growth observed in enrolment, “progress in learning has been slow” (Angrist et al., 2021). Alternative estimates looking back over a longer time horizon finds little improvement in school quality over 50 years in developing countries (Le Nestour et al., 2022). It has been incredibly difficult to find concrete examples of countries or even places within countries that have seen rapid sustained progress in improving learning. Whilst it is possible to find examples of success, its just as easy to find examples of failure. Take for example the 2014–15 USAID reading programme in Nigeria. The programme cost US$ 9 million, was based on evidence on what works to improve early grade reading, and followed a common protocol of scripted lesson guides, teacher coaching, and pupil books. By the end of the project the 5655 age 8 grade 2 children in treatment schools could only identify 5 correct letter sounds per minute, and 5 correct words per minute. The share of children able to answer 4 out of 5 reading comprehension questions correctly was 0.0% (RTI International, 2015).

A key concern around feasibility is scalability. The need for scalable interventions is being increasingly recognised, yet popular approaches to prioritisation based on cost-effectiveness analysis continue to neglect what works at scale. For example the GEAAP Best Buys report, works out which interventions have produced the most learning per dollar spent, but includes many interventions that haven’t actually been shown rigorously to work at scale. This matters when effect sizes for many interventions shrink dramatically as they scale. This is particularly the case for those that involve widespread difficult behaviour change. Researchers working with high quality NGOs to implement reading interventions at small scale can get impressive gains in learning. But trying to get thousands of teachers across the country to change their daily behaviour without the same level of expert support and attention is much less likely to success. Attempts to replicate the same effect at national scale in government systems often fails. Examples abound, including for example hiring new contract teachers (Bold et al., 2018), a mother-tongue literacy program (Kerwin and Thornton, 2021), and promoting growth mindsets (Ganimian, 2020). By contrast, other interventions are inherently more scalable - that are relatively easy to implement. These include more ‘logistical’ tasks - such as building schools, extending the length of the school day, removing tuition fees, providing scholarships, providing universal free meals, or some kinds of educational technology (Crawfurd et al., 2022b).

## 5 Total spending levels are not fixed but respond to good spending opportunities

Rational policymakers choose to spend more on things they think are going to be impactful. For years now economists have been arguing that spending doesn’t matter in education, incentives do. Smart policymakers have caught on, and limited such perceived “wasteful” spending. If we don’t make the economic case for investing in education, policymakers will rationally choose not to invest. Much cost-effectiveness analysis proceeds from a position that budgets are limited. Yet as the case of PEPFAR shows, a politically attractive investment case can galvanise new funding (Sandefur, 2023). Similarly, success in achieving free primary education by removing fees has only come where countries have been able to find new money for schools, not in reallocating education funds from somewhere else (Crawfurd et al., 2021).

Ministers need a plan. Setting ambitious learning goals might help to spur action, but it does not provide a clear plan for action. Whilst some early grade reading programs have been somewhat successful, effects are often smaller for larger national scale programmes. The steps to improving learning scale rely on scarce implementation capacity. By contrast, removing artificial barriers to secondary school enrolment, chiefly exam pass thresholds and tuition fees, can be done at close to the stroke of a pen. Countries already send capitation (per pupil) grants to schools to pay for materials. Replacing fees simply requires increasing those grants. Where insufficient schools exist, major school building programmes have been successful even in weak governance environments with low implementation capacity, from Indonesia to Afghanistan. Where sufficient schools do already exist, making school compulsory has been successful in increasing enrolment.

## 6 Conclusion

The SDG education agenda is broad, but progress requires focus where returns are clearest. The economic and social benefits of completing the access agenda are well-established. Abolishing fees and barriers to enrollment, especially at secondary level, can expand access rapidly if financed. This approach is also more politically feasible than trying to transform classroom practices to improve learning, which has proven difficult to scale up.

No one disputes the importance of learning, but access may be the surer path to progress. Removing barriers to enrollment delivers a concrete plan ministers can implement and see results. The steps required to improve learning at scale are far less clear.

Efforts to improve pedagogy can of course progress alongside expanding access, but should not divert focus and resources from the most achievable strategy with clearest returns - getting all children through a full cycle of education by removing barriers to enrollment. This foundation will enable progress across the wider SDG education agenda. This access agenda can be broadly conceived, including looking at not just enrolment but ensuring children can attend for a full school day and receive a free meal whilst at school.

Governments and the donors that advise them should focus on simple reforms that are hard to get wrong, with a reliable pay-off, not on difficult reforms that are hard to get right and have an uncertain pay-off.

## Declarations of Competing Interest

None.
